# Diamine vapor treatment of viscoelastic graphene oxide liquid crystal for gas barrier coating

**DOI:** 10.1038/s41598-021-88955-5

**Published:** 2021-05-04

**Authors:** Seung Eun Choi, Sung-Soo Kim, Eunji Choi, Ji Hoon Kim, Yunkyu Choi, Junhyeok Kang, Ohchan Kwon, Dae Woo Kim

**Affiliations:** 1 Department of Chemical and Biomolecular Engineering, Yonsei University, Yonsei-ro 50, Seodaemun-gu, Seoul, 03722 Republic of Korea; 2 Carbon Composite Materials Research Center, Korea Institute of Science and Technology, 92 Chudong-ro Bongdong-eup, Wanju-gun, Jeollabuk-do 55324 Republic of Korea

**Keywords:** Materials science, Nanoscale materials, Structural materials

## Abstract

A layered graphene oxide/ethylenediamine (GO/EDA) composite film was developed by exposing aqueous GO liquid crystal (GOLC) coating to EDA vapor and its effects on the gas barrier performance of GO film were systematically investigated. When a GO/EDA coating with a thickness of approximately 1 μm was applied to a neat polyethylene terephthalate (PET) film, the resultant film was highly impermeable to gas molecules, particularly reducing the gas permeance up to 99.6% for He and 98.5% for H_2_ in comparison to the neat PET film. The gas barrier properties can be attributed to the long diffusion length through stacked GO nanosheets. The EDA can crosslink oxygen-containing groups of GO, enhancing the mechanical properties of the GO/EDA coating with hardness and elastic modulus values up to 1.14 and 28.7 GPa, respectively. By the synergistic effect of the viscoelastic properties of GOLC and the volatility of EDA, this coating method can be applied to complex geometries and EDA intercalation can be spontaneously achieved through the scaffold of the GOLC.

## Introduction

Impermeable barrier coatings are essential in various applications, such as food packaging, electronic devices, energy storage, membrane, and hydrogen storage^1–4^. Among the various impermeable materials, graphene is an ideal barrier due to its high mechanical strength, chemical stability, and most importantly, its impermeability to all gas molecules even for helium and hydrogen^5^. Graphene is also intrinsically flexible, stretchable, and transparent owing to its atomic-scale thickness^6,7^. Considering all these advantages, single-layer graphene synthesized by chemical vapor deposition (CVD) was deposited on polymeric substrates or stacked in the form of a multilayer graphene film^8^. However, the transfer of CVD graphene onto a large area without the formation of cracks is challenging, and the generation of point defects and grain boundaries is unavoidable during the CVD process^9^. In addition, the metal catalyst (usually Cu, Ni, or alloy foil) needs to be removed by an acidic etchant such as FeCl_3_, raising critical issues in terms of environmental impact and fabrication costs^6^. Therefore, although CVD graphene could be an ideal gas barrier material, the deposition of defect-free graphene on a large scale onto various substrate geometries remains challenging.

As an alternative, graphene nanosheets including few-layer graphene flakes, graphene oxide (GO), and reduced graphene oxide (rGO) have been frequently hybridized with polymers, inorganic particles, and organic molecules^10–12^. The incorporation of impermeable fillers with high aspect ratios effectively enhances the gas barrier performance by increasing the diffusion length of permeating gas molecules^13^. Abundant oxygen-containing groups of graphene derivatives are also beneficial to the functionalization of the graphene surface, to enhance the solubility in organic solvents, and to improve the miscibility of graphene with polymer matrix^14^. More importantly, the preparation of graphene derivatives, particularly GO, can be scalable to satisfy the demand of nanofillers for composite preparation^4,15–19^. Nonetheless, the high loading of graphene fillers in the polymer matrix is hindered by the aggregation and restacking induced by van der Waals interactions between graphene sheets^20–22^.

The stacked structure of GO sheets in the form of a laminated film on the polymeric substrate has been suggested as a promising method to solve this problem. While the GO coating is permeable to water molecules, it can block the permeation of all gas molecules^23^. In addition, the GO layer becomes impermeable to both water and gas molecules after chemical reduction^24^. To achieve the barrier properties of stacked GO films, several fabrication methods have been suggested such as spin-coating^25^, spray-coating^26^, vacuum filtration^27,28^, bar-coating^29,30^, slot-die coating^31^, and layer-by-layer (LBL) assembly^3,32^. LBL assembly is particularly effective owing to its numerous advantages; it allows the easy control of the GO coating thickness in the nanometer-scale with highly aligned GO nanosheets, and can be applied to complex geometries with excellent gas barrier performances^32–34^. However, the fabrication procedure is laborious, therefore, the development of an efficient fabrication procedure is imperative to meet the industry requirements^35^.

Herein, a layered GO/EDA composite coating with gas barrier properties was achieved by exposing aqueous GO liquid crystal (GOLC) coating to EDA vapor, utilizing the volatility of EDA. The diffusion of EDA was highly facilitated into the interlayer and scaffold of GOLC and the amounts of intercalated EDA molecules can be precisely controllable by adjusting the exposure time. The intercalation mechanism and the EDA structure between GO nanosheets were thoroughly examined to the exposure time of EDA vapor. In addition, we performed a systematic analysis of the changes in mechanical properties and gas permeation of the GO/EDA coatings according to the amount of EDA in the designed film.

## Results and discussion

**Figure 1:**
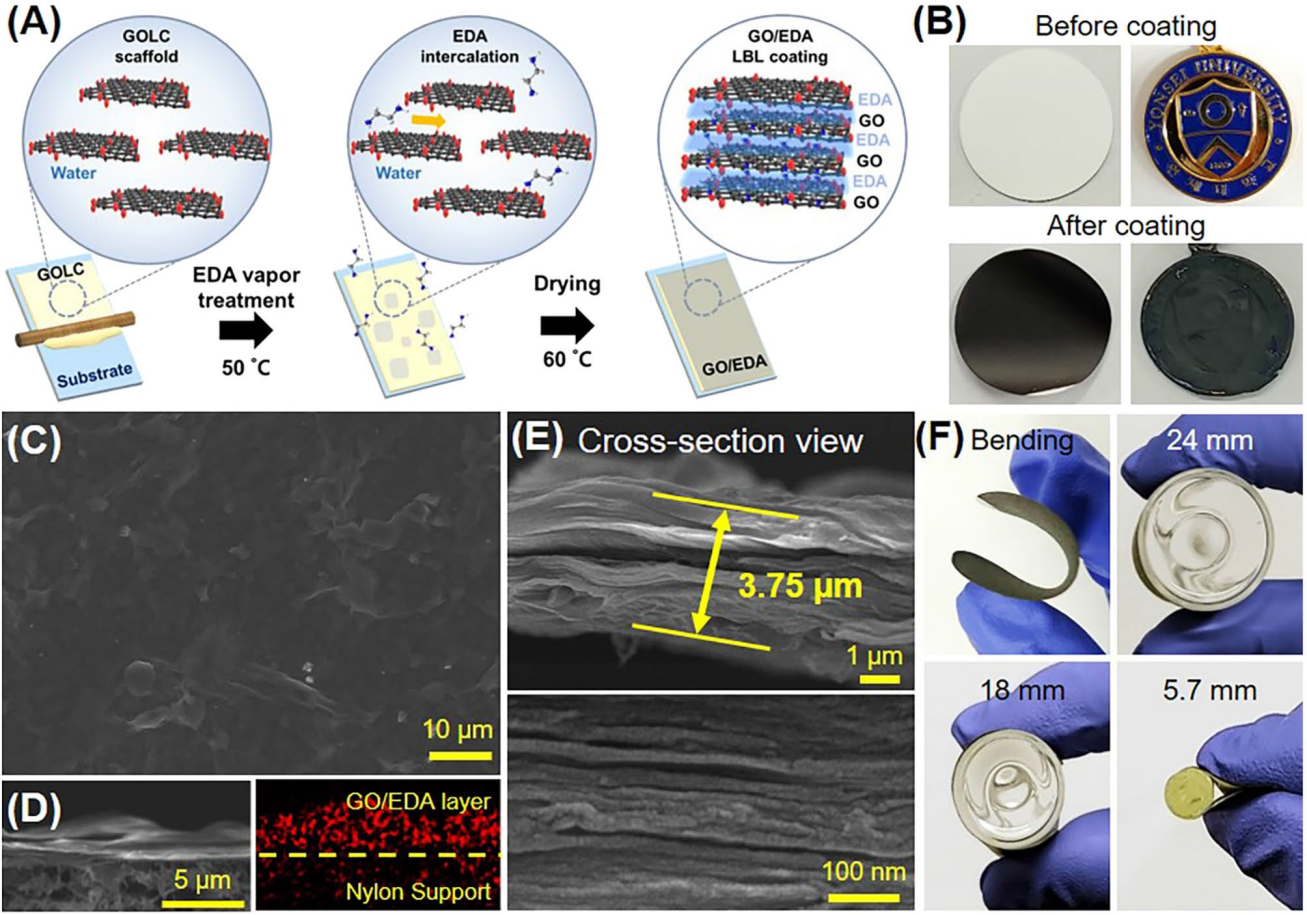
(**A**) Schematic illustration of GO/EDA coating procedure on the target substrate. (**B**) Photographic images of a nylon substrate and a souvenir before and after GO/EDA coating. (**C**,**D**) Top-view, and cross-sectional SEM images of a GO/EDA coating on a nylon substrate. The right image of (**D**) is EDS mapping image for nitrogen. (**E**) Magnified cross-sectional SEM images of GO/EDA coating. (**F**) Bending test of GO/EDA coated a nylon substrate at several bending diameters. The EDA exposure time was 1 h.

The procedure for GO/EDA coating on a target surface is portrayed in Fig. 1A and the corresponding photographic images are shown in Supplementary Fig. S1. GOLC with a concentration of 30 mg/mL was prepared by dispersing GO in water via sonication, which is a typical method to induce the lyotropic LC phase of GO in an aqueous solvent^36^. The gel-like GO solution was distributed on a target substrate using a bar-coater. The thickness of GOLC is adjustable by changing the gap between the bar and the substrate. In this study, the thickness of the GOLC coating was fixed at 180 μm. A porous nylon substrate (pore size of 0.1 μm) was used as a substrate to demonstrate the feasibility of our coating method on surfaces with complex structures. A 75-μm-thick PET film was used for the gas permeation test (Supplementary Fig. S2). The GOLC coated substrate was placed in the center of a petri dish and 0.5 mL of 75% EDA aqueous solution was spread around it. Then, it was kept in an oven at 50 °C for the desired time. Higher temperatures are inappropriate because the water in the GOLC coating may dry too quickly. After a certain time under EDA vapor treatment, the GO/EDA coated substrate was dried at 25 °C to remove excess water, resulting in the formation of a dense GO/EDA coating. The GO/EDA coating can be applied to various objects, such as a more complex object like a school souvenir as shown in Fig. 1B. We also want to emphasize the advantage of using EDA vapor for the treatment of GOLC. While our previous work based on the EDA solution immersion is effective for the treatment of flat type coating or small objective, using EDA solution treatment has the limitation in applying the coating in large-size objects such as hydrogen cylinders owing to the possibility of the GOLC coating delamination during the immersion process^33^. Therefore, EDA vapor treatment was chosen for the crosslinking reagent, because it is beneficial for applying in scale-up coating and reducing laborious and time-consuming process.

To verify the feasibility of our method to produce a uniform GO/EDA coating layer, SEM images were acquired from the GO/EDA coating with 1 h exposure to the EDA vapor (Fig. 1C,D). A uniform GO/EDA coating layer was observed on the nylon substrate (Fig. 1C). The GO sheets were stacked parallel to the substrate (Fig. 1D,E) because the shear coating process aligned the GOLC scaffold in parallel with the substrate^33^. The EDS mapping of nitrogen marked with red color showed the homogeneous distribution of amine groups, indicating uniform diffusion of EDA molecules into the inner regions of the GOLC. A round morphology was observed between the GO sheets in the magnified cross-sectional SEM images of GO/EDA (Fig. 1E), while the edge of neat GO sheets was sharp (Supplementary Fig. S3). This denoted the presence of EDA surrounding each GO layer, which is stably adhered to the graphene surface even in the high vacuum conditions during the SEM measurements. As shown in the bending test, the GO/EDA coating on the nylon substrate was highly bendable and could be bent to the 5.7 mm diameter of a glass rod (Fig. 1F). This is due to the intrinsic flexible nature of graphene, and this flexibility could be critical in applications such as flexible electronics, food packaging, and gas storage.

**Figure 2:**
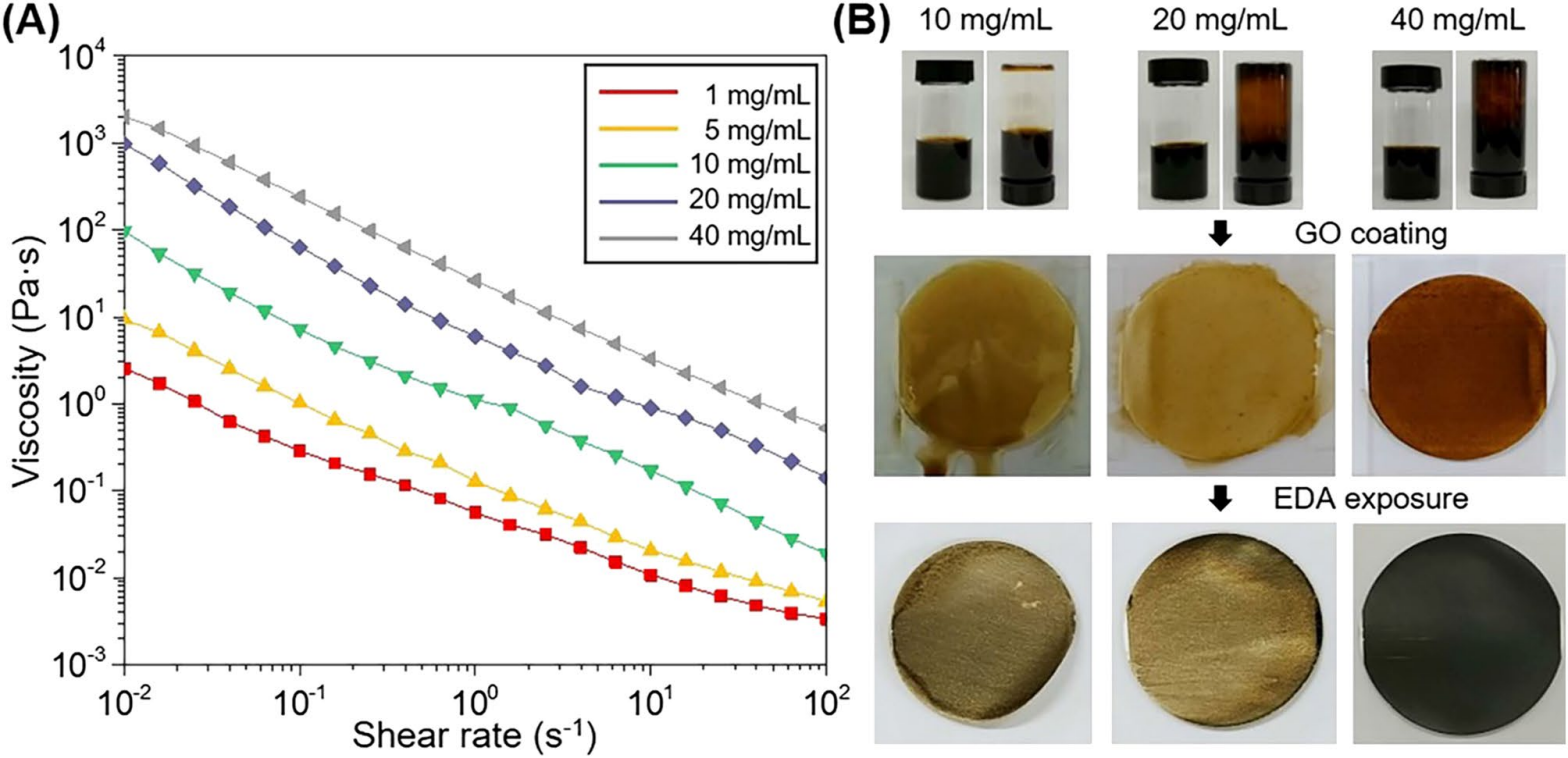
Viscoelastic rheological properties of the GO solution. (**A**) Viscosity of GO solution as a function of shear rate and concentration. (**B**) GO/EDA coating quality depending on the concentration of the GO solution. The EDA exposure time was 1 h.

The viscoelastic rheological properties are essential for uniform coating of GOLC on complex geometries by using the shear-coating method and to preserve the shape of the GOLC coating during EDA vapor exposure^33,36,37^. Primarily, the viscoelastic properties of GOLC were investigated by measuring the GOLC viscosity as a function of the shear rate and concentration (Fig. 2A). The viscosity of GOLC gradually increased with the concentration from 1 to 40 mg/mL. Particularly, the viscosity of GOLC reached 1 × 10^3^ and 3 × 10^3^ Pa∙s for 20 and 40 mg/mL, respectively, in static conditions. However, the viscosity of 40 mg/mL GOLC spontaneously decreased to 9 × 10^–1^ Pa∙s at a shear rate of 100 s^−1^, indicating gel-like and shear-thinning viscoelastic behavior; therefore, the viscosity is highly influenced by the shear rate^33,38,39^.

The coating process was also conducted with GO solutions of 10, 20, and 40 mg/mL (Fig. 2B). The photographic images of the vials containing the GO solutions show that the 10 mg/mL solution was watery, while GO solutions of 20 mg/mL and 40 mg/mL adhered to the wall of the vials even after turning the vial upside down. This indicates that a solid-like behavior of the GO solutions can be observed at the higher concentrations, as demonstrated by the rheology study shown in Fig. 2A. To reveal the importance of a high viscosity GOLC, the nylon substrates with GOLC coating were tilted at 45° as shown in the second row of the images of Fig. 2B. As expected, the GOLC coating was only preserved for the 40 mg/mL solution, while for lower concentrations the coating flowed to the bottom. Therefore, a higher concentration of GOLC produces better quality GO/EDA films by preserving the shape of the GOLC coating during EDA exposure (the third row of the images of Fig. 2B). This observation is particularly important when GO/EDA coating is applied to objects with a three-dimensional morphology and tilted surfaces such as gas storage tanks.

**Figure 3:**
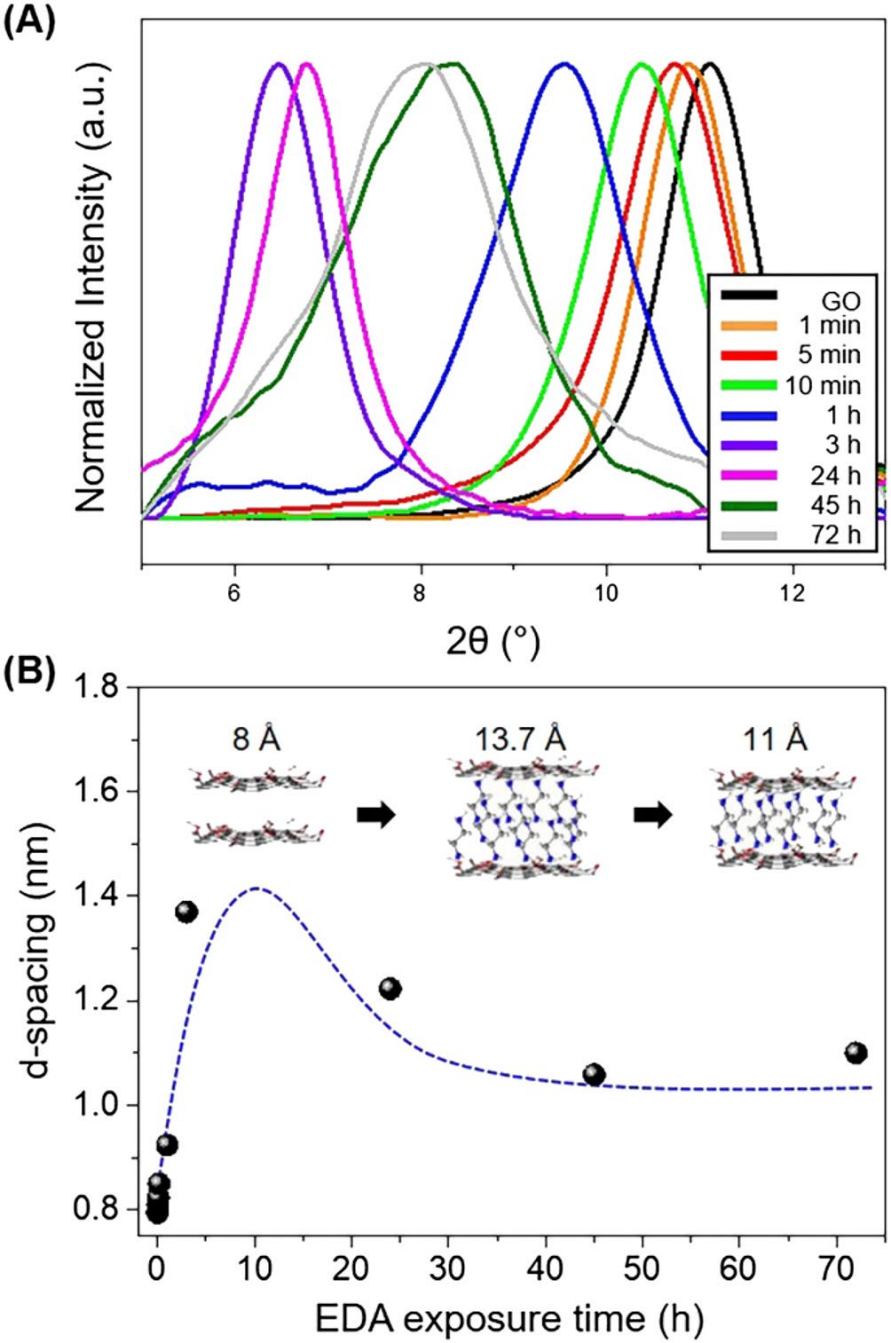
(**A**) XRD patterns of GO/EDA as a function of EDA vapor exposure time. (**B**) Corresponding d-spacing of GO/EDA.

XRD analysis was conducted to examine the intercalation of EDA into the GOLC scaffold and its interlayer structure (Fig. 3, Supplementary Fig. S4). Figure 3A displays the XRD patterns of GO/EDA coating and Fig. 3B summarizes the corresponding interlayer distance. The interlayer distance of GO was 7.95 Å, which is in agreement with the literature^23^. The interlayer distance of the GO/EDA coating was 8.24 Å after 5 min of EDA exposure, indicating the spontaneous intercalation of EDA into the GOLC scaffold. The value increased to 9.24 Å after 1 h of EDA exposure and further increased to 13.7 Å after exposure for 3 h. We want to emphasize that the expanded interlayer spacing is not by the presence of water because all samples were fully dried at 60 °C for 24 h before XRD measurement and neat GO did not show similar expansion of interlayer spacing. The interlayer distance was reduced to 13.1 Å after 1 d of exposure. After 3 d of exposure, the interlayer distance was further decreased and saturated to 11.0 Å at a longer EDA exposure time of 72 h, which is the typical interlayer distance of GO cross-linked with EDA^40^. Considering the length of an EDA molecule (around 3.7 Å) and the thickness of a graphene layer (3.4 Å)^21^, it can be assumed that a double layer of EDA molecules exists between the GO sheets at the low EDA exposure times as described in the inserted scheme of Fig. 3B because excessive EDA can diffuse into the scaffold of GOLC spontaneously. The rearrangement of the EDA molecules may occur at exposure times over 3 h as some of the EDA is possibly consumed in the formation of crosslinking with oxygen-containing groups of GO or unreacted EDA molecules are removed from the composite system with time^33,40^. We believe the reduction of GO is not the main reason for the thickness decrease because most of the oxygen-containing groups are detected by XPS even after EDA treatment, which we will deliberate in detail below. Similarly, the increasing thickness of the GO/EDA coating with increased EDA exposure time was observed in the SEM images of the GO/EDA coating (Supplementary Fig. S3). The influence of EDA exposure time on the mechanical and gas barrier properties of the GO/EDA coating will be discussed later.

**Figure 4:**
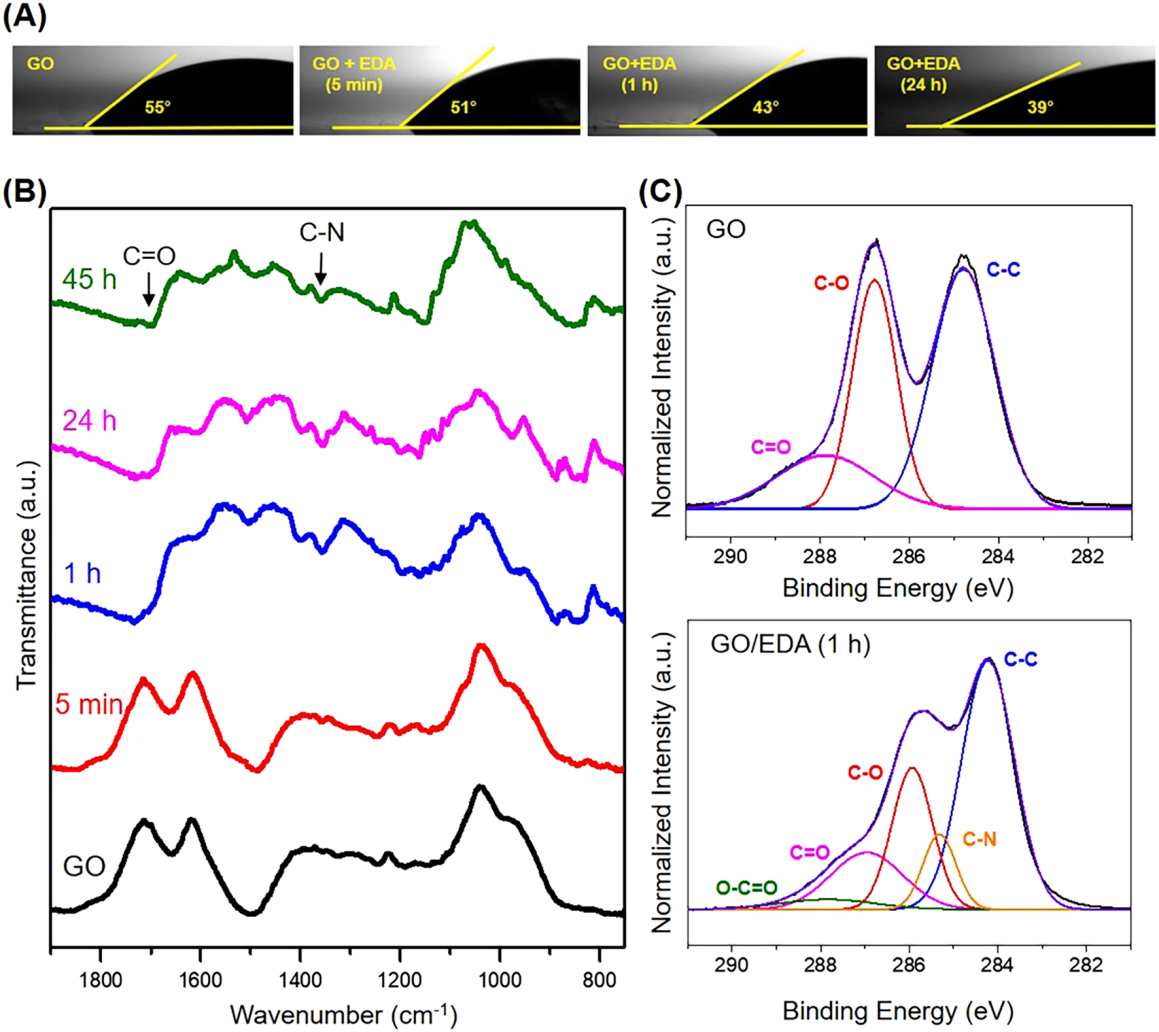
(**A**) Water contact angles and (**B**) FT-IR spectra of GO and GO/EDA film depending on the EDA exposure time. (**C**) XPS C1s spectra of GO and GO/EDA. EDA was exposed for 1 h.

The chemical structure of the GO/EDA coating was examined using water contact angle measurements, XPS, FT-IR (Fig. 4), and Raman spectroscopy (Supplementary Fig. S5). The water contact angle of GO was 55° and gradually decreased to 39° with increasing EDA exposure time (Fig. 4A). The more hydrophilic surface of the GO/EDA coating can be ascribed to the amine groups of EDA molecules that are bonded to the surface of GO as considering the protonation of amine groups in the presence of water^40^. The FT-IR spectra confirmed the variation of functional groups in GO and GO/EDA coatings, particularly the C=O stretching of amide (1710 cm^−1^) and C–N stretching (1340 cm^−1^) (Fig. 4B). The C=O stretching peak became clear as the EDA exposure time increased. This tendency was observed because the amine groups of the EDA and the oxygen-containing groups of GO form an amide bond^33^. At the same time, the C–N stretching peak began to appear, which implies the presence of EDA molecules in the GO/EDA coating.

Figure 4C shows the C1s spectra of GO and GO/EDA coating. Typical oxygen-containing groups of C–O (286.8 eV) and C=O (287.9 eV) were detected in the circumstance of the GO coating. A new C–N peak appeared at 285.3 eV after EDA treatment due to the presence of both unreacted amine groups and the amide bonding of EDA. Also, the intensity of the C–O and C=O peaks in the XPS spectra of GO/EDA (1 h) was relatively lower compared to that of the GO peaks because of the partial reduction of oxygen groups in the presence of the alkaline EDA^40^. The reduction mechanism can be a nucleophilic ring-opening reaction in the presence of amines, which means that the amine groups disconnect the oxygen moieties and remain sp^2^ hybridized carbon bonding^41^. This partial reduction reaction between GO and EDA was reconfirmed by the Raman spectra, which showed an increased I_D_/I_G_ ratio as the EDA exposure time increased (Supplementary Fig. S5). It is known that the I_D_/I_G_ peak ratio of GO increases by chemical reduction due to the generation of dense and small sp^2^ carbon domains^21^. Based on the aforementioned results, we conclude that EDA crosslinks the GO sheets and also partially reduces GO, thus, the reduction becomes significant at longer EDA exposure times. However, we want to notice that the EDA treatment conditions are not enough to fully reduced GO into rGO as reflected by the presence of oxygen groups in GO/EDA samples (Fig. 4B,C).

**Figure 5:**
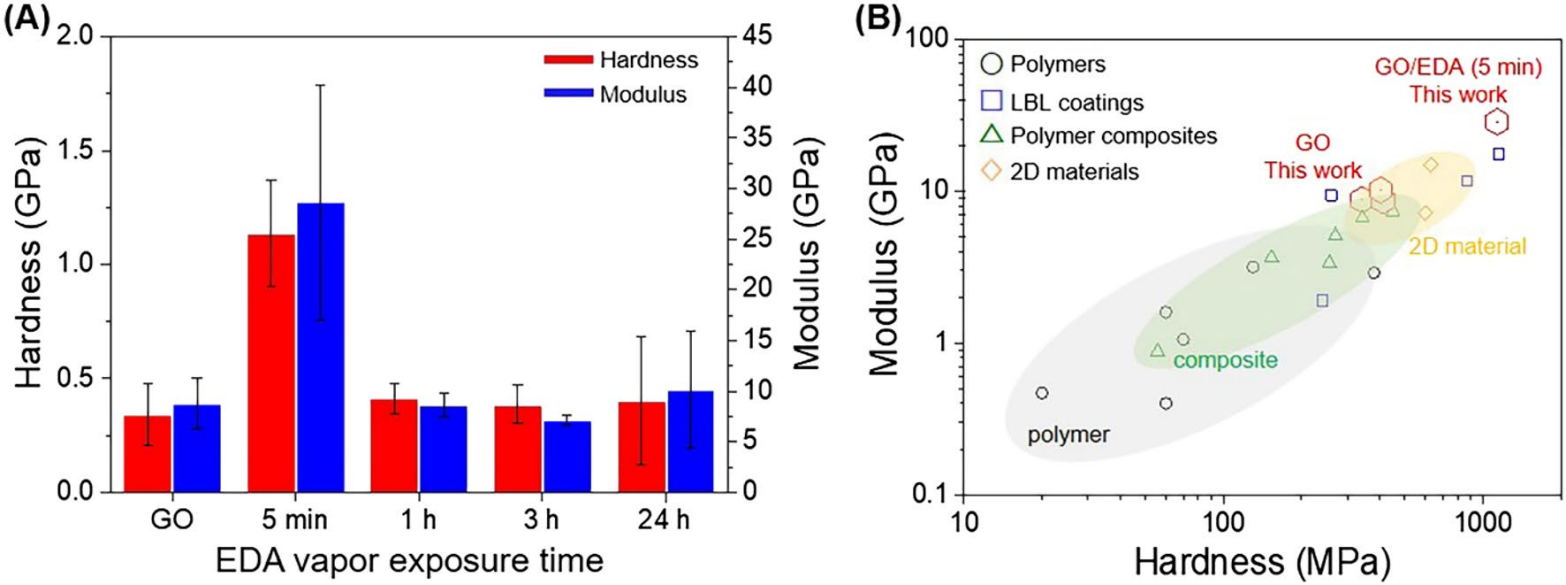
(**A**) The hardness and modulus of GO/EDA coatings measured by the nanoindentation technique and the average values were obtained from 3 to 5 points for each sample. (**B**) Mechanical properties comparison between GO/EDA coating and various coating materials.

The mechanical properties of the GO/EDA coating with various EDA exposure times were measured using the nanoindentation method and compared with a neat GO film (Fig. 5A). The hardness of GO was 0.34 GPa and that for the GO/EDA coatings was 1.14, 0.41, 0.39, and 0.40 GPa for 5 min, 1 h, 3 h, and 24 h of exposure, respectively. The elastic modulus of GO was 8.78 GPa and that of the GO/EDA coatings was 28.7, 8.68, 7.13, and 10.2 for EDA exposure times of 5 min, 1 h, 3 h, and 24 h, respectively. Both hardness and elastic modulus of the GO/EDA coating were relatively higher than those of GO. The highest values of hardness and elastic modulus were observed at EDA vapor exposure for 5 min. It appeared that the mechanical properties of GO were highly enhanced when low amounts of EDA were present to crosslink adjacent GO sheets. In contrast, excessive EDA hindered the crosslinking by decorating the surface of GO as evidenced by the SEM image shown in Fig. 1E.

The mechanical properties of the GO/EDA films (marked with red hexagons) are higher than polymers, polymer composite films, LBL films, and two-dimensional (2D) materials as summarized in Fig. 5B and Supplementary Table S1^4,33,42–55^. The hardness and modulus of polymer were reported as in the range of 20–380 MPa and 0.4–2.9 GPa. The hardness and the modulus of polymer composite films (green triangles) were in the range of 56–1510 MPa and 0.26–7.35 GPa, respectively. As expected, LBL film (blue squares) had better mechanical properties as 240–1150 MPa for hardness and 1.9–17.64 GPa for modulus. Neat 2D materials such as MoS_2_ showed hardness and modulus between 600–630 MPa and 7.2–15 GPa, respectively. Although various coating materials generally express adequate mechanical properties, the mechanical properties of GO/EDA coating are excellent particularly at 5 min of EDA exposure time with 1135 MPa for hardness and 28.7 GPa for modulus, which is beneficial for the utilization of the GO/EDA coating in high-pressure conditions such as high-pressure H_2_ storage tank.

**Figure 6:**
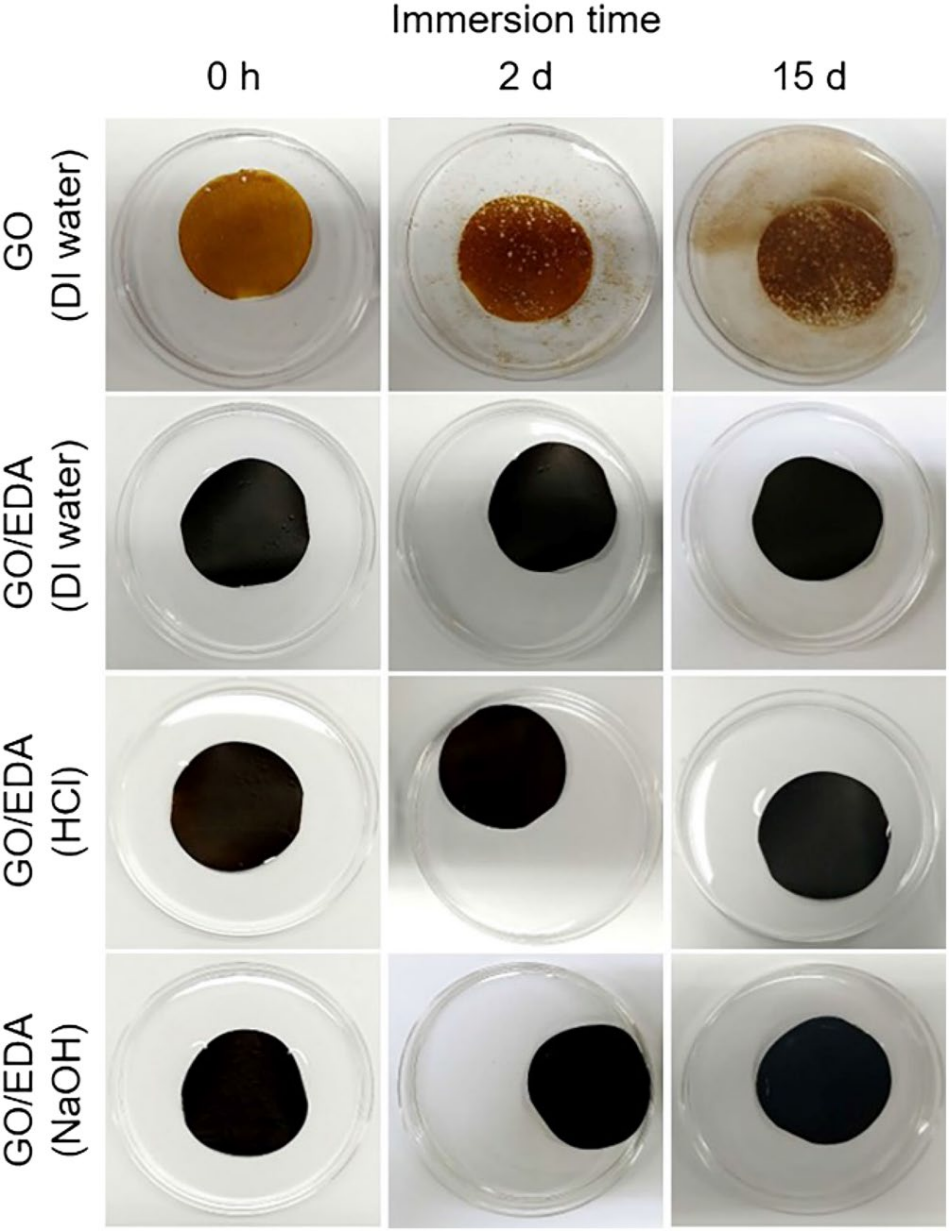
Stability of GO and GO/EDA coatings in aqueous solvents including water. The exposure time of EDA was 1 h. The concentration of HCl and NaOH solutions was 1 M.

While GO coating is known as an impermeable gas barrier^23^, the stability of GO in water is one of the critical problems associated with the use of the GO film in humid conditions or aqueous solvents^56^. Thereby, the stability of the GO/EDA coating in water, acidic solution, and basic solution was tested as shown in Fig. 6. The concentration of acidic and basic solutions was 1 M, which was controlled by the volumes of HCl and NaOH, respectively. A neat GO film on the nylon substrate was unstable and easily re-dispersed in water due to the abundant oxygen functional groups on the surface of GO^57^. In contrast, GO/EDA coating on the nylon support was stable in water, acidic conditions, and basic conditions even after 15 days of immersion. The stability of the GO/EDA coating can be attributed to the crosslinking between GO sheets and EDA molecules as well as enhanced π–π interaction between partially reduced GO sheets as discussed in Fig. 4.

**Figure 7:**
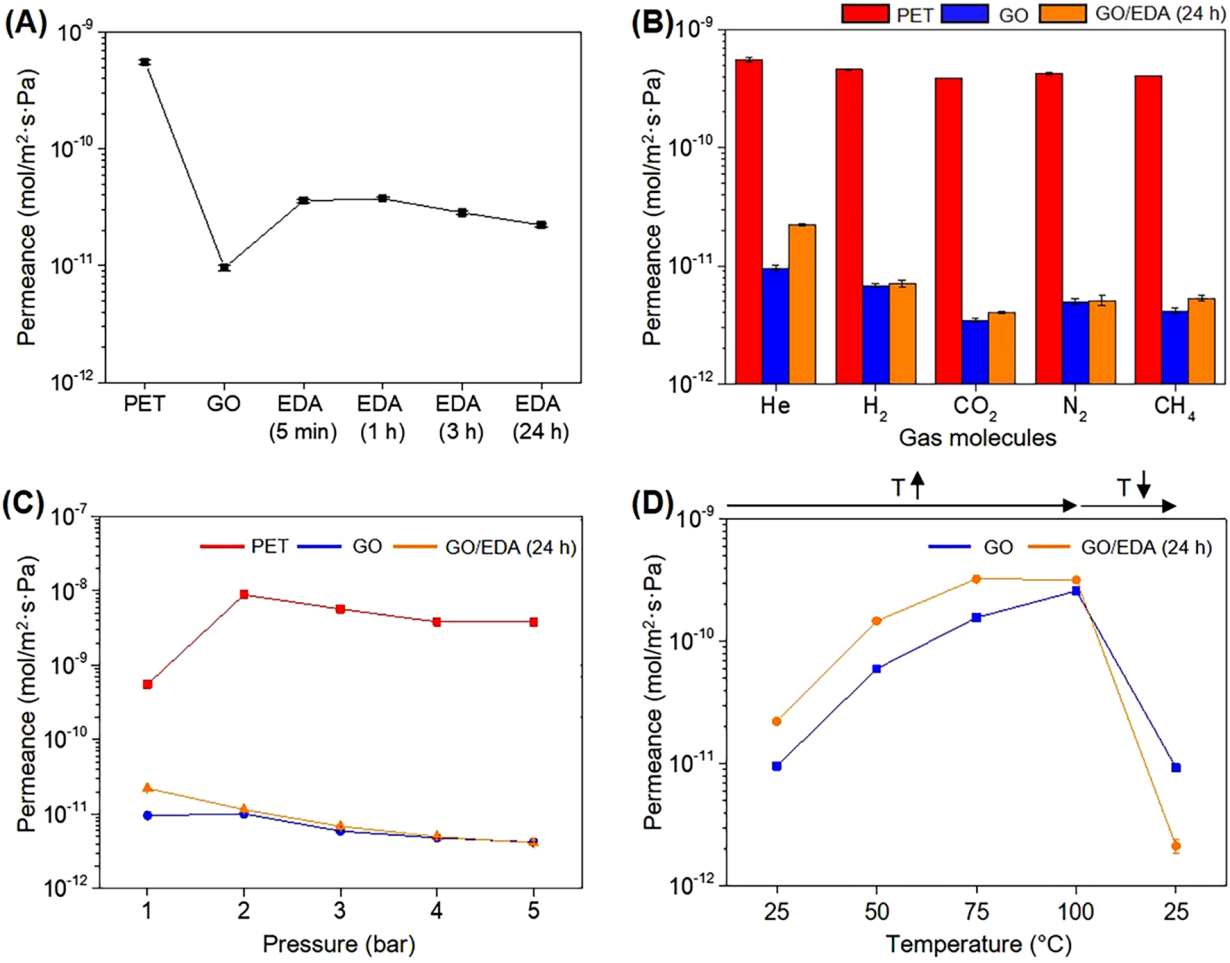
(**A**) He gas permeance of PET and GO/EDA depending on EDA exposure time. The thickness of GO was approximately 1 μm. (**B**) Gas permeances of neat PET, GO coated PET, and GO/EDA (24 h) coated PET. (**C**) He gas permeance of PET, GO coated PET, and GO/EDA (24 h) coated PET as a function of pressure. (**D**) He gas permeance of GO and GO/EDA (24 h) coated PET as a function of temperature. Each point represents the average of a minimum of three measurements.

The gas permeance of GO/EDA coating was measured using a time lag method with the equipment illustrated in Supplementary Fig. S6 (Fig. 7). A 75-μm-thick PET film, one of the most widely used polymers for packaging, was used as a substrate film and the thickness of GO and GO/EDA coating was approximately 1–1.5 μm (Supplementary Fig. S7). First, the variation of the He permeance with EDA exposure time was measured to verify the influence of EDA amounts on the gas permeance of the GO/EDA coating and to evaluate the best GO/EDA structure to block gas permeation (Fig. 7A). (number) denotes the EDA exposure time of GO/EDA sample. The transmembrane pressure was 1 bar and the temperature was 25 °C. As previously reported^23,33^, the GO film was highly impermeable to He with a permeance of 9.58 × 10^–12^ mol/m^2^ s Pa, which represents a 98.3% reduction to the neat PET film (5.54 × 10^–10^ mol/m^2^ s Pa). The He permeances for GO/EDA (5 min), (1 h), (3 h), and (24 h) were 3.60 × 10^–11^, 3.75 × 10^–11^, 2.85 × 10^–11^, and 2.23 × 10^–11^ mol/m^2^ s Pa, respectively. Note that the permeance of GO/EDA coating is slightly higher than that of GO coating possibly due to the expanded interlayer spacing in the presence of EDA. Nonetheless, all GO/EDA coatings can enhance barrier properties of PET film, and the best He barrier performance was achieved at 24 h of EDA exposure time, showing a 96.0% reduction compared to the neat PET film.

The neat PET film, neat GO, and GO/EDA (24 h) coating were further tested with several gases including He, H_2_, CO_2_, N_2_, and CH_4_ at 1 bar and 25 °C (Fig. 7B). The neat PET film was permeable to all tested gas molecules showing a permeance in the range of 3.87 × 10^–10^ mol/m^2^ s Pa (CO_2_)–5.54 × 10^–10^ mol/m^2^ s Pa (He). Neat GO films were highly impermeable to all gas molecules, showing permeance in the range of 3.46 × 10^–12^ mol/m^2^ s Pa (CO_2_)–9.58 × 10^–12^ mol/m^2^ s Pa (He), which represents a minimum 98.5% reduction for H_2_ and up to 99.1% reduction for CO_2_ compared to PET. Interestingly, the GO/EDA (24 h) film was also showed its highly impermeable property to different gas molecules with permeance ranging from 2.23 × 10^–11^ to 7.12 × 10^–12^ mol/m^2^ s Pa. The reduction degree compared to PET was approximately 98.5% for H_2_ and 99.0% for CO_2_ and the permeance reduction was particularly dramatic for gas molecules lager than He and H_2_. As indicated by the permeance tendency of all five gas molecules, the GO/EDA (24 h) film possessed a similar gas barrier performance to that of the neat GO film, which is known to have excellent gas barrier performance^23^.

He permeance of GO/EDA (24 h) was also examined by increasing the transmembrane pressure from 1 to 5 bar at room temperature (25 °C) as shown in Fig. 7C. While the He permeance of the neat PET film increased from 5.54 × 10^–10^ to 3.06 × 10^–9^ mol/m^2^ s Pa for 1 and 5 bar, respectively, the He permeance of GO/EDA (24 h) decreased from 2.23 × 10^–11^ to 4.11 × 10^–12^ mol/m^2^ s Pa for 1 and 5 bar, respectively, and a similar tendency was detected for neat GO. Therefore, the GO/EDA coating is particularly effective to block gas molecules at high pressure with a 99.3% reduction of He permeance at 5 bar compared to neat PET.

The influence of temperature on the He permeance of the GO/EDA (24 h) film was investigated by increasing the temperature from 25 to 100 °C and back to 25 °C (Fig. 7D). The He permeance of neat GO gradually increased from 9.58 × 10^–12^ mol/m^2^ s Pa at 25 °C to 2.59 × 10^–10^ mol/m^2^ s Pa at 100 °C as indicated by the blue line in Fig. 7D. The GO/EDA (24 h) film also showed a similar tendency with increased He permeance from 2.23 × 10^–11^ to 3.19 × 10^–10^ mol/m^2^ s Pa at 25 °C and 100 °C, respectively, which can be attributed to activated gas diffusion at high temperature, as previously reported for a laminated GO film^33,58,59^. After cooling to 25 °C, the He permeance of the GO coated film (9.31 × 10^–12^ mol/m^2^ s Pa) was nearly the same as the value before heat treatment (9.58 × 10^–12^ mol/m^2^ s Pa). In contrast, the He permeance of GO/EDA (24 h) was further reduced to 2.14 × 10^–12^ mol/m^2^ s Pa after cooling. Interestingly, this value is even lower than that of neat GO film, emphasizing that the additional heat treatment of GO/EDA (24 h) is effective in further enhancing the gas barrier properties of the GO/EDA coating.

To understand the origin of the enhanced gas barrier properties of GO/EDA (24 h) after heat treatment, additional experiments were conducted as shown in Supplementary Fig. S8. XRD patterns, Raman spectra, FT-IR spectra, and XPS C1s spectra of GO/EDA (24 h) after heat treatment at 100 °C demonstrated a better alignment of the GO nanosheets. This can be explained by the rearrangement of EDA between GO nanosheets, as a narrow interlayer spacing can be formed by the reduction of some of the oxygen-containing groups of GO (Supplementary Fig. S8). In addition, excessive and unreacted EDA molecules can be possibly removed from the composite film. Therefore, the enhanced gas barrier performance of GO/EDA (24 h) after heat treatment can be attributed to the abundantly arranged EDA molecules in the narrow nanochannels of GO, which hinder the diffusion of gas molecules by steric hindrance and hydrogen bonding interaction of polar molecules^3,33,60^. In contrast, the low amount of EDA can increase the space of the nanochannels, resulting in faster gas permeation than neat GO, which happens in the case of GO/EDA (5 min) shown in Fig. 7A. Therefore, controlling the interlayer spacing of GO and the amount of intercalated EDA is critical for achieving gas barrier performance.

The barrier performance of the GO/EDA (24 h) film is comparable with previous graphene-based gas barrier coatings as shown in Supplementary Table S2^3,4,10,13,24,32,61–63^. Since experimental conditions such as coating thickness, coating methods, and coating materials are different, it is hard to compare the gas barrier performance directly. Nevertheless, the comparison could be helpful to understand the effectiveness of the GO/EDA coating to block gas molecules. The permeance reduction of He and H_2_ was particularly organized because they are the smallest molecules and highly permeable and because there is a growing demand for the hydrogen storage tank with light materials. Simply mixed graphene/polymer coating shows 82–99.9% reduction of H_2_ permeance when applied onto PET or nylon substrates^3,4,10,13,24,32,62–64^. As expected, graphene-coatings prepared by LBL methods and neat rGO coating have reported high gas barrier performances with approximately 41.1–99.9% reduction of H_2_ and He permeance^24,64^. While our GO/EDA coating procedure is simple and readily applicable for coating various objects, the coating surpasses the performances of the aforementioned graphene polymer composites coating with a reduction of 99.6% and 98.5% in the permeances of He and H_2_, respectively.

## Conclusions

We demonstrated that a highly concentrated GOLC system is effective to prepare composite materials based on graphene/small molecules. Since GOLC facilitates the diffusion of EDA through the macropores of its scaffolds and EDA is highly volatile even at low temperatures, intercalation and crosslinking of EDA between graphene sheets can be achieved spontaneously. Furthermore, the viscoelastic properties of GOLC are beneficial to coat well-aligned graphene sheets on the target surface. The intercalated EDA acts as a cross-linker and as a reducing agent; thus, the GO/EDA coating is highly stable in water, acids, and bases. The gas barrier performance of the GO/EDA film can be enhanced than that of neat GO film when the GO/EDA (24 h) coating was heat-treated at 100 °C. The heat-treated GO/EDA (24 h) film showed a permeance reduction of 99.6% and 98.5% for He and H_2_, respectively, at atmospheric conditions compared to a neat PET film. We believe this coating strategy can provide insight into the preparation of other 2D material-based multifunctional composite films and this method is also useful for the fabrication of hydrogen storage containers.

## Methods

### GO synthesis

Graphite (≤ 20 μm, 99% pure, Sigma Aldrich) was oxidized using a modified Hummer’s method by following the literature^27^. Graphite (4 g) and KMnO_4_ (12 g) were mixed with 150 mL of sulfuric acid (98%). After stirring the solution for 5 h at 35 °C, deionized (DI) water and hydrogen peroxide (H_2_O_2_) were added in sequence, in an ice bath. The solution was then filtrated with a cellulose filter to remove any remaining acidic solution and washed several times with DI water. The filtrated GO was re-dispersed with DI water and freeze-dried to obtain GO powder.

### Fabrication of the GO/EDA film

GO (3 g) was dispersed in 100 mL of DI water by ultrasonication for 3 h to prepare 30 mg/mL of GO solution. At the concentration, LC phase was observed by using polarized optical microscopy^33^. A nylon substrate (Sterlitech, 0.1 µm pore diameter) and a polyethylene terephthalate (PET) film (75 μm thickness) were coated with GOLC to a thickness of 180 µm. The thickness was controlled using a bar-coater (Yoshimitsu, YBA-7). The substrates coated with GOLC were exposed to EDA vapor generated from an EDA solution (75%, 0.5 mL) for different exposure times (1, 5, and 10 min, 1, 24, 45, and 72 h) at 50 °C. The volatility of EDA accelerates its migration from the EDA solution into the GOLC coating. After exposure to the EDA vapor, the GO/EDA coated substrates were dried at 25 °C for 24 h to remove the excess water.

### Gas permeance measurement

Gas permeance was measured by a constant volume and differential pressure method by following the literature^64,65^, which is one of the most widely used techniques for measuring gas permeance. Films were placed on a stainless-steel mesh support and fixed using epoxy glue before the measurement. The sample cell was divided into two parts, the film side and mesh side. The mesh was under vacuum while the target gas was flowing on the other side with a flow rate of 20 sccm. The pressure of feed gas was controlled by using a backpressure regulator and a gas cylinder regulator. The vacuum of the permeation side was measured by using a vacuum gauge (Teledyne Hastings Instruments, HVG-2020B). The gas permeance was calculated with the following equation:

$$\text{Permeance}=\frac{{V}_{c}}{R*T*{P}_{a}*A}\frac{dp}{dt}$$

where V_C_ is the chamber volume of the vacuum side (98.5 mL), A is the effective film area (m^2^), P_a_ is the atmospheric pressure or transmembrane pressure (Pa), dp/dt is the pressure variation of the chamber on the vacuum side per unit of time, and R is the gas constant. T is the temperature (K), which was controlled using a convection oven.

### Characterization

Scanning electron microscopy (SEM) images were obtained using a Field Emission Scanning Electron Microscopy (FE-SEM, JEOL, 7610f-plus). The viscosities of GO solutions were measured with a cone plate geometry (diameter: 40 mm; angle: 1° 58′ 55″) and a Peltier plate temperature system of a discovery hybrid rheometer (DHR-3, TA Instruments) at 25 °C. Flow sweep tests were conducted with shear rates between 10^−5^ and 100 s^−1^ and a fixed truncation gap of 55 μm. X-ray diffraction (XRD) patterns were obtained using a SmartLab (Rigaku, wavelength: 1.54 Å) diffractometer. Water contact angle images were measured using an Attension Theta Lite (Biolin Scientific) optical tensiometer. Fourier-transform infrared (FT-IR) spectra were obtained using a Spectrum 100 (Perkin Elmer, USA). X-ray photoelectron spectroscopy (XPS) measurement was performed using a K-alpha (Thermo Fisher Scientific) with a Cu(Kα) beam source (wavelength 1.5406 Å). Nanoindentation tests were conducted using a nanoindentation system (Nano Indenter XP, MTS). Raman spectra were obtained using a LabRam Aramis (Horriba Jovin Yvon) with a 532 nm laser.

## Supplementary Information

Supplementary Information 1.
