# Supplementary material for: Diamine vapor treatment of viscoelastic graphene oxide liquid crystal for gas barrier coating
Source: Sci Rep. 2021 May 4;11:9518. doi: 10.1038/s41598-021-88955-5 (PMC8096969; doi:10.1038/s41598-021-88955-5)
Supplement: Supplementary file 1 — Supplementary Information 1. [file 41598_2021_88955_MOESM1_ESM.docx]

**Supporting Information**

**Diamine vapor treatment of viscoelastic graphene oxide liquid crystal for gas barrier coating**

Seung Eun Choi^1^, Sung-Soo Kim^2^, Eunji Choi^1^, Ji Hoon Kim^1^, Yunkyu Choi^1^, Junhyeok Kang^1^, Ohchan Kwon^1^, Dae Woo Kim^1,*^

^1^Department of Chemical and Biomolecular Engineering, YONSEI University, Yonsei-ro 50, Seodaemun-gu, Seoul, (03722), Republic of Korea

^2^Carbon Composite Materials Research Center, Korea Institute of Science and Technology, 92 Chudong-ro Bongdong-eup, Wanju-gun, Jeollabuk-do 55324, Republic of Korea

^*^ Corresponding author. Tel: 82-2 2123-2745. E-mail: [audw1105@yonsei.ac.kr](mailto:audw1105@yonsei.ac.kr) (Dae Woo Kim)


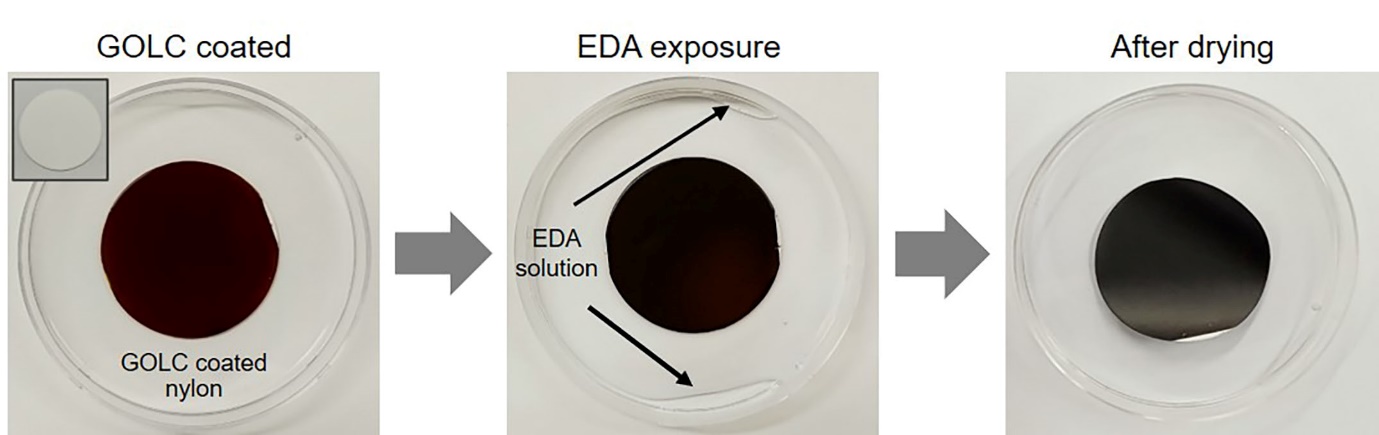


**Figure S1.** Pictures of GO/EDA coating procedure. The inset is a neat nylon substrate.


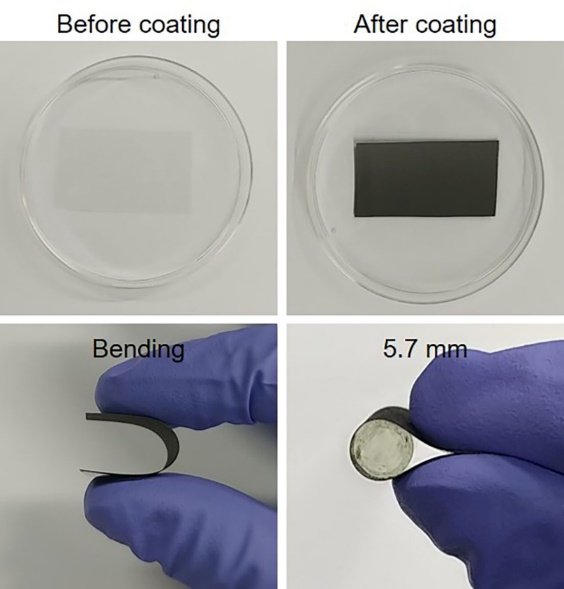


**Figure S2.** Photographic images of a PET substrate before and after GO/EDA coating and bending test of the coated film. The GO/EDA coating on the PET substrate was bendable at the 5.7 mm diameter of a glass rod. The EDA exposure time was 1 h.


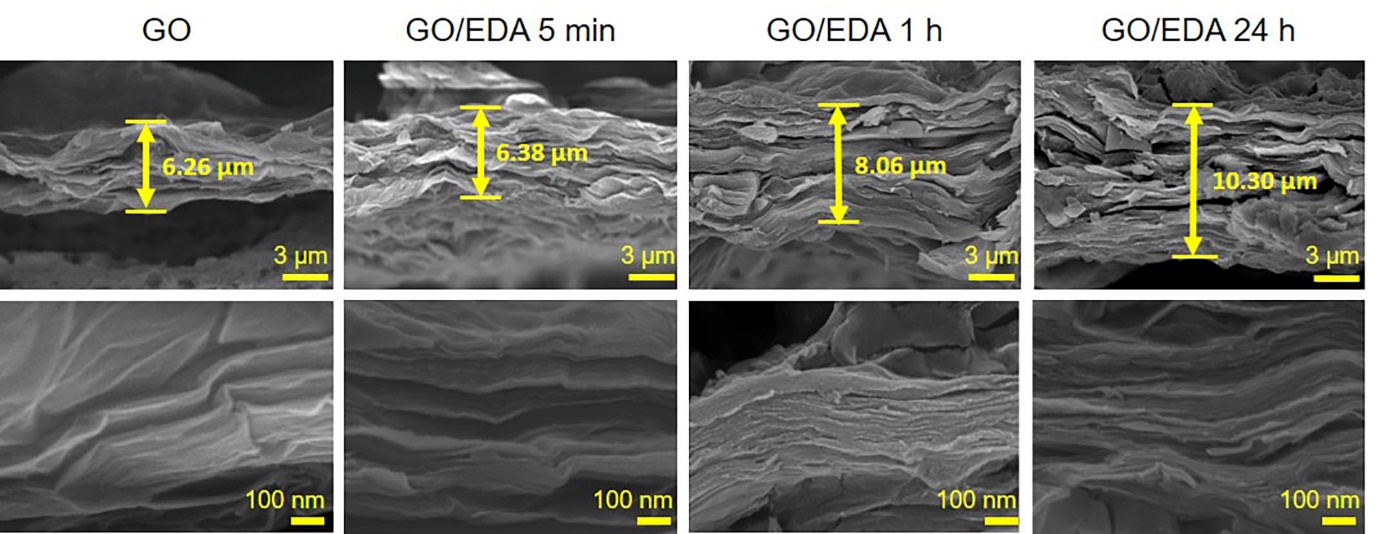


**Figure S3.** Thickness variation of GO/EDA coating on nylon substrate depending on EDA exposure time in low magnification and high magnification (top and bottom images, respectively). From the left, neat GO, GO/EDA with exposure of 5 min, 1 h, and 24 h, respectively. While the same amounts of GOLC was applied, the thickness of the GO coating increased with EDA addition, corresponding to XRD spectra showing increased d-spacing of GO by EDA vapor treatment. The sharp edges of laminated GO sheets became smoother as the EDA exposure time increased.


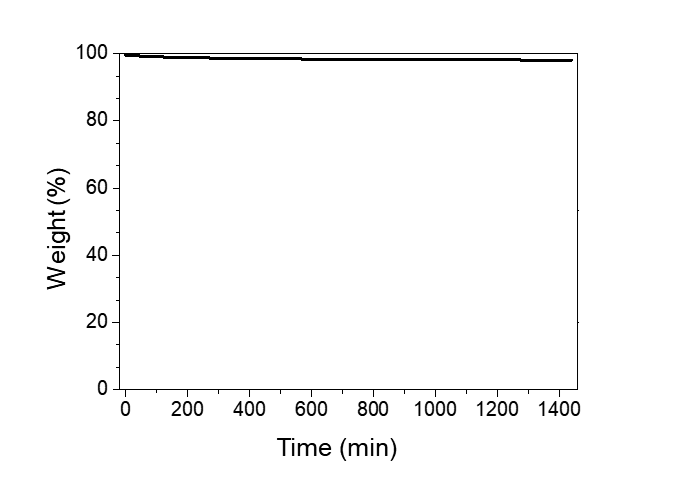


**Figure S4.** TGA plot of GO/EDA (24 h). The weight variation of the sample was measured at 50°C for 24 h. The weight loss is not significant, indicating that intercalated EDA is stable in the interlayer of GO.


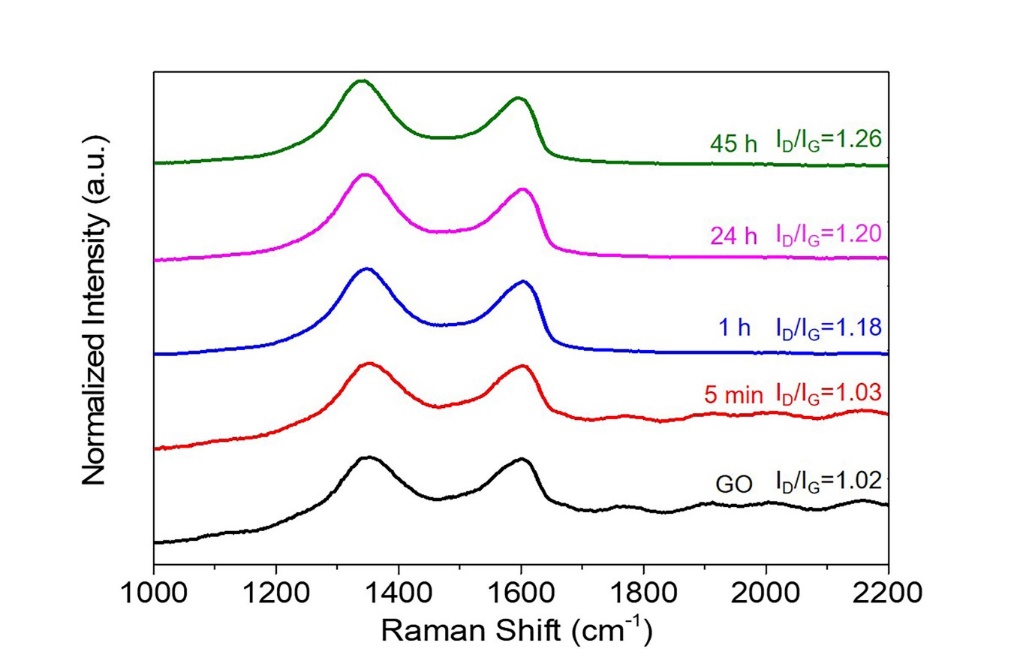


**Figure S5.** Raman spectra of GO and GO/EDA coatings depending on EDA exposure time. The I_D_/I_G_ ratio of GO increased with the exposure time of EDA vapor, indicating the chemical reduction of GO by diamine at 50°C.


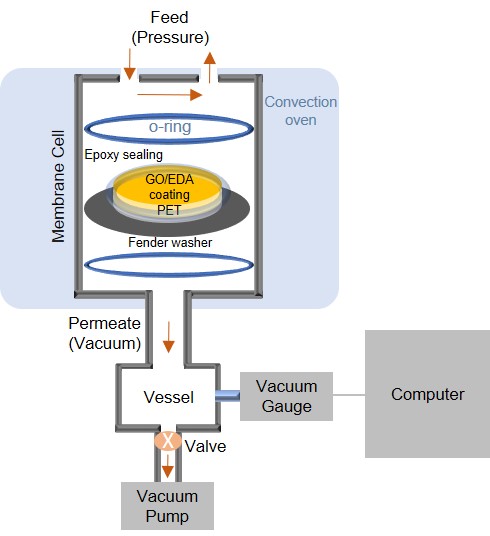


**Figure S6.** Schematic illustration of gas permeance measurement equipment.


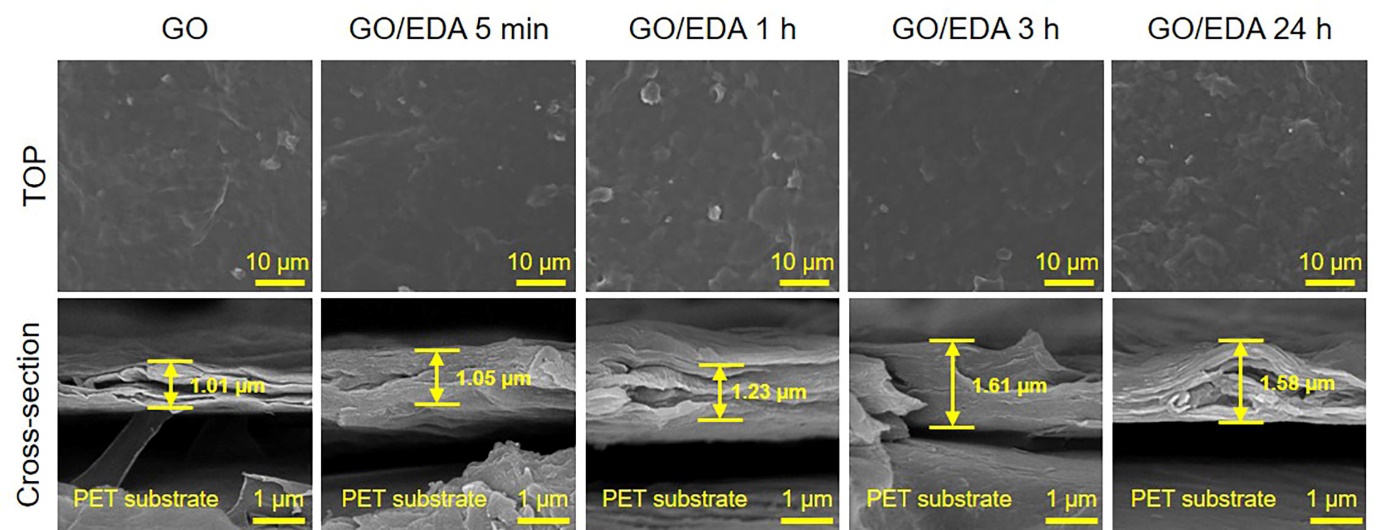


**Figure S7.** Thickness variation of GO/EDA film on PET substrate depending on EDA exposure time. GO/EDA film detachment was inevitable during the preparation of SEM samples using liquid nitrogen and mechanical fracturing.


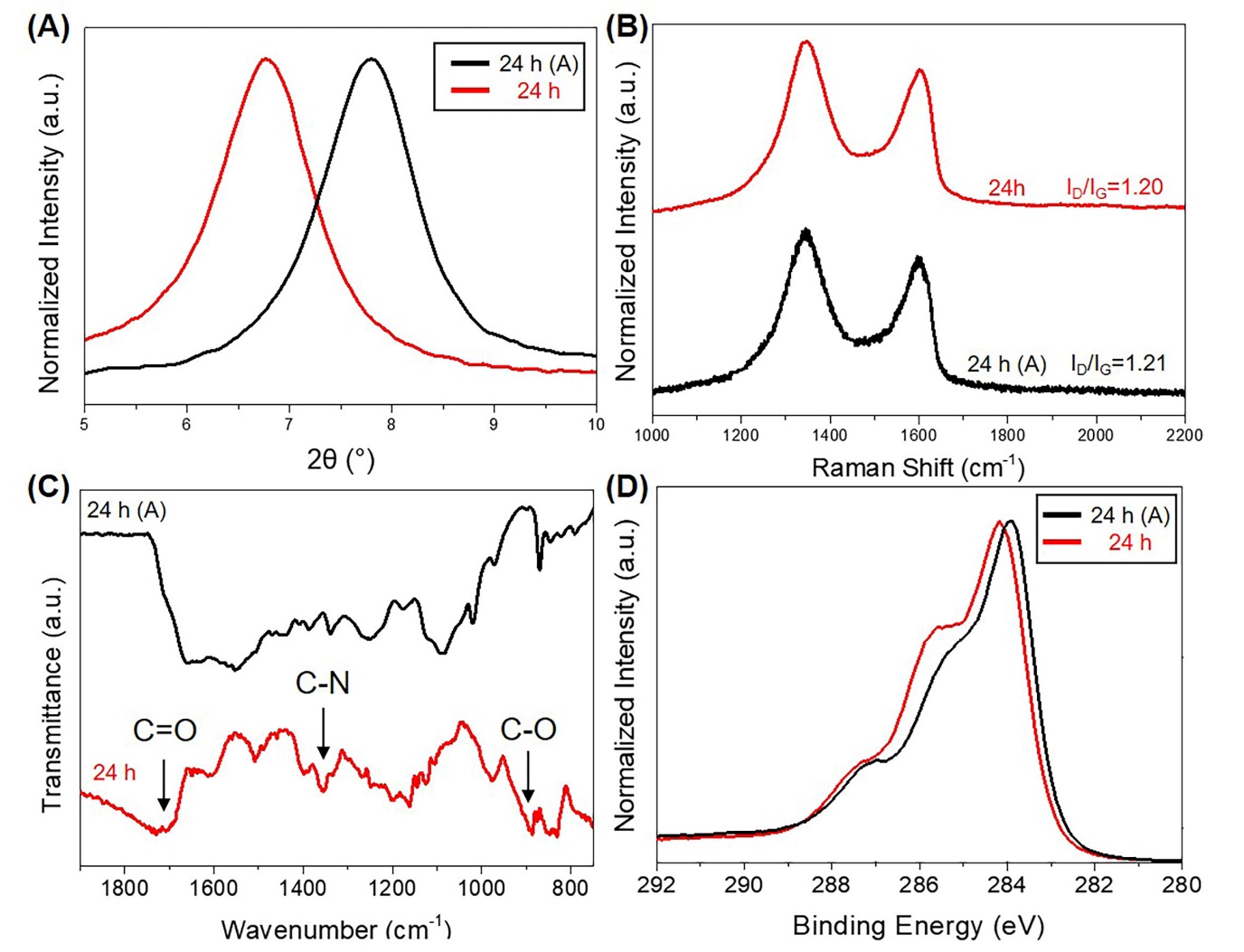


**Figure S8.** (**A**) XRD patterns of GO/EDA (24 h) and GO/EDA (24 h) after treatment at 100°C for 2 h. (**B**) Raman spectra of GO/EDA (24 h) before and after treatment at 100°C for 2 h. (**C**) FT-IR spectra of GO/EDA (24 h) before and after treatment at 100°C for 2 h. (**D**) XPS C 1s spectra of GO/EDA (24 h) before and after the treatment at 100°C for 2 h.

The XRD patterns indicate the formation of narrow and more ordered interlayer spacing of GO/EDA after the heat treatment. The composition ratio of oxygen of GO/EDA (24 h) decreased due to the thermal treatment, inducing a partial reduction of GO as observed in Supplementary Fig. S6C,D. The Raman spectra of GO/EDA (24 h) are identical regardless of the heat treatment because the structure variation of GO occurs mainly by the removal of oxygen groups rather than recovery of sp^2^ carbon domains.

**Table S1.** Mechanical properties of various coating materials. The values were evaluated by a nanoindentation technique.

| **Material** | **Type** | **Hardness (MPa)** | **Modulus (GPa)** | **Ref** |
| --- | --- | --- | --- | --- |
| Silicate | Polymer composite | 343 | 6.646 | 1 |
| PVA  (0.6 % of GO) | Polymer composite | 55.7 | 0.885 | 2 |
| PMMA-matrix  (0.6 % of GO) | Polymer composite | 153.1 | 3.65 | 2 |
| Epoxy (6 wt % of GNP) | Polymer composite | 256 | 3.36 | 3 |
| PVA  (1 % of RGO) | Polymer composite | 270 | 5.08 | 4 |
| FPBO  (17 wt % of nRGO) | Polymer composite | 450 | 7.35 | 5 |
| EVOH (15 % of BA & 5 % of GO) | Polymer composite | 1510 | 0.26 | 6 |
| PVA/GO | LBL | 1150 | 17.64 | 7 |
| Cellulose/GO | LBL | 870 | 11.70 | 8 |
| PEI-modified GO/PAA | LBL | 240 | 1.9 | 9 |
| RGO/EDA | LBL | 260 | 9.4 | 10 |
| MXene | 2D material | 630 | 15 | 11 |
| MoS_2_ | 2D material | 600 | 7.2 | 12 |
| PVA | Polymer | 70 | 1.06 | 4 |
| PC | Polymer | 380 | 2.9 | 13 |
| PET | Polymer | 130 | 3.2 | 14 |
| HDPE | Polymer | 60 | 1.6 | 15 |
| LDPE | Polymer | 20 | 0.47 | 15 |
| PU | Polymer | 60 | 0.4 | 16 |
| GO | 2D material | 342 | 8.78 | **This work** |
| GO+EDA (5 min) | LBL | 1135 | 28.7 | **This work** |
| GO+EDA (1 h) | LBL | 412 | 8.68 | **This work** |
| GO+EDA (24 h) | LBL | 403.3 | 10.17 | **This work** |

Polyvinly alcohol (PVA), Poly(methyl methacrylate) (PMMA), Graphene nanoplatelets (GNP), reduced graphene oxide (RGO), fluorinated-polybenzoxazole (FPBO), Graphene oxide with a sub-micrometer diameter (nGO), poly(vinyl alcohol-co-ethylene) (EVOH), Boric acid (BA), poly(ethyleneimine) (PEI), Polyacrylic acid (PAA), polycarbonate (PC), polyethylene terephthalate (PET), high-density polyethylene (HDPE), low-density polyethylene (LDPE), polyurethane (PU), graphene oxide (GO), Ethylenediamine (EDA)

**Table S2.** Comparison of He and H_2_ barrier performance of GO/EDA film for previous graphene-based barrier coatings.

| **Fabrication method** | **Coating material** | **Coating thickness** | **Substrate type** | **Substrate thickness** | **Gas type** | **Permeance reduction** | **Ref** |
| --- | --- | --- | --- | --- | --- | --- | --- |
| GO single phase/  Rod-coating or  spray coating | Reduced graphene oxide  (reducing agent:  hydroiodic acid) | 200 nm | PET | 12 µm | He | 99.9% | 17 |
|  |  | 30 nm |  |  | H_2_ | 99.8% |  |
| Mixed solution/  Spray coating | Hexylamine (HA)/  reduced graphene oxide | 10.2 µm  (13 wt% of HA) | Nylon | 3.1 mm | H_2_ | 82.0% | 18 |
| Mixed solution/  Spray coating | Graphene oxide/ octadecylamine/ maleic anhydride grafted polypropylene (MAPP) | 23 µm  (60 wt% of MAPP) | Nylon | 0.08 mm | H_2_ | 94.1% | 19 |
| Mixed solution/  Spray coating | Boric acid (BA)/  poly(vinyl alcohol-co-ethylene)/ graphene oxide | 21 µm  (15 % of BA;  5 wt% of GO) | Nylon | 70 µm | H_2_ | 96.1% | 6 |
| Mixed solution/  Spray coating | Ethylenediamine/ graphene oxide | 11 µm | Nylon | 3.2 mm | H_2_ | 88.4% | 20 |
|  | Triethylenetetramine/ graphene oxide | 10.8 µm |  |  |  | 93.0% |  |
| Mixed solution/  Spray coating | Polyvinyl alcohol/  graphene oxide | 2.56 µm  (9 ml of composite solution) | PET | 110 µm | H_2_ | 96.0% | 21 |
| Layer-by-Layer/  Dip coating | Polyethyleneimine modified graphene oxide | 20 nm  (24 bilayers) | PET | 117 µm | H_2_ | 79.1% | 22 |
|  | Poly(sodium 4-styrenesulfonate) modified graphene oxide | 270 nm  (24 bilayers) |  |  |  | 97.0% |  |
| Layer-by-Layer/  Dip coating | Polyethyleneimine/  graphene oxide (0.05 wt%) | 100 nm  (20 bilayers) | PET | 180 µm | H_2_ | 41.1% | 23 |
| Layer-by-Layer/  Dip coating | Poly(ethyleneimine)/  graphene oxide | 185 nm (50 bilayers) | PET | 100 µm | He | 99.3% | 24 |
|  |  |  |  |  | H_2_ | 99.9% |  |
| Layer-by-Layer/  Bar coating | Ethylenediamine/  graphene oxide | 1~1.5 µm | PET | 75 µm | He | 99.6% | **This work** |
|  |  |  |  |  | H_2_ | 98.5% |  |

**Supplementary References**

1. Dhakal, H. N., Zhang, Z. Y. & Richardson, M. O. W. Nanoindentation behaviour of layered silicate reinforced unsaturated polyester nanocomposites. *Polym*. *Test*. **25**, 846-852, (2006).

2. Das, B., Eswar Prasad, K., Ramamurty, U. & Rao, C. N. Nano-indentation studies on polymer matrix composites reinforced by few-layer graphene. *Nanotechnology* **20**, 125705, (2009).

3. King, J. A., Klimek, D. R., Miskioglu, I. & Odegard, G. M. Mechanical properties of graphene nanoplatelet/epoxy composites. *J*. *Appl*. *Polym*. *Sci*. **128**, 4217-4223, (2013).

4. Sharma, B., Shekhar, S., Gautam, S., Sarkar, A. & Jain, P. Nanomechanical analysis of chemically reduced graphene oxide reinforced poly (vinyl alcohol) nanocomposite thin films. *Polym*. *Test*. **70**, 458-466, (2018).

5. Shin, S., Kim, J. S., Kim, S. J., Kim, D. W. & Jung, H. Polybenzoxazole/graphene nanocomposite for etching hardmask. *J*. *Ind*. *Eng*. *Chem*. **75**, 296-303, (2019).

6. Li, X., Bandyopadhyay, P., Guo, M., Kim, N. H. & Lee, J. H. Enhanced gas barrier and anticorrosion performance of boric acid induced cross-linked poly(vinyl alcohol-co-ethylene)/graphene oxide film. *Carbon* **133**, 150-161, (2018).
7. Zhao, X. *et al.* Alternate multilayer films of poly(vinyl alcohol) and exfoliated graphene oxide fabricated via a facial layer-by-layer assembly. *Macromolecules* **43**, 9411-9416, (2010).
8. Tang, L., Li, X., Du, D. & He, C. Fabrication of multilayer films from regenerated cellulose and graphene oxide through layer-by-layer assembly. *Prog*. *Nat*. *Sci*. **22**, 341-346, (2012).

9. Wang, N., Ji, S., Zhang, G., Li, J. & Wang, L. Self-assembly of graphene oxide and polyelectrolyte complex nanohybrid membranes for nanofiltration and pervaporation. *Chem*. *Eng*. *J*. **213**, 318-329, (2012).
10. Kim, D. W., Kim, H., Jin, M. L. & Ellison, C. J. Impermeable gas barrier coating by facilitated diffusion of ethylenediamine through graphene oxide liquid crystals. *Carbon* **148**, 28-35, (2019).
11. Hatter, C. B., Shah, J., Anasori, B. & Gogotsi, Y. Micromechanical response of two-dimensional transition metal carbonitride (MXene) reinforced epoxy composites. *Compos*. *B*. *Eng*. **182**, (2020).

12. Panich, N., Wangyao, P., Hannongbua, S., Sricharoenchai, P. & Sun, Y. Effect of polytetrafluoroethylene doping on tribological property improvement of MoS_2_ nano-thin films on Ti-substrate. *Rev*. *Adv*. *Mater*. *Sci*. **16**, 88-95 (2007).
13. Fang, T. & Chang, W. Nanoindentation characteristics on polycarbonate polymer film. *Microelectron*. **35**, 595-599, (2004).
14. Flores, A. *et al.* Structure formation in poly(ethylene terephthalate) upon annealing as revealed by microindentation hardness and X-ray scattering. *Polymer* **46**, 9404-9410, (2005).

15. Gracias, D. & Somorjai, G. Continuum force microscopy study of the elastic modulus, hardness and friction of polyethylene and polypropylene surfaces. *Macromolecules* **31**, 1269-1276 (1998).

16. Gupta, T. K., Singh, B. P., Dhakate, S. R., Singh, V. N. & Mathur, R. B. Improved nanoindentation and microwave shielding properties of modified MWCNT reinforced polyurethane composites. *J*. *Mater*. *Chem*. *A* **1**, (2013).

17. Su, Y. *et al.* Impermeable barrier films and protective coatings based on reduced graphene oxide. *Nat*. *Commun*. **5**, 4843, (2014).
18. Bandyopadhyay, P. *et al.* Hexylamine functionalized reduced graphene oxide/polyurethane nanocomposite-coated nylon for enhanced hydrogen gas barrier film. *J*. *Membr*. *Sci*. **500**, 106-114, (2016).

19. Li, X., Bandyopadhyay, P., Nguyen, T. T., Park, O. & Lee, J. H. Fabrication of functionalized graphene oxide/maleic anhydride grafted polypropylene composite film with excellent gas barrier and anticorrosion properties. *J*. *Membr*. *Sci*. **547**, 80-92, (2018).
20. Bandyopadhyay, P., Nguyen, T. T., Li, X., Kim, N. H. & Lee, J. H. Enhanced hydrogen gas barrier performance of diaminoalkane functionalized stitched graphene oxide/polyurethane composites. *Compos*. *B*. *Eng*. **117**, 101-110, (2017).

21. Layek, R. K., Das, A. K., Park, M. U., Kim, N. H. & Lee, J. H. Layer-structured graphene oxide/polyvinyl alcohol nanocomposites: dramatic enhancement of hydrogen gas barrier properties. *J*. *Mater*. *Chem*. *A* **2**, (2014).

22. Liu, H. *et al.* Layer-by-layer assembled polyelectrolyte-decorated graphene multilayer film for hydrogen gas barrier application. *Compos*. *B*. *Eng*. **114**, 339-347, (2017).

23. Yang, Y. H., Bolling, L., Priolo, M. A. & Grunlan, J. C. Super gas barrier and selectivity of graphene oxide-polymer multilayer thin films. *Adv*. *Mater*. **25**, 503-508, (2013).

24. Pierleoni, D. *et al.* Selective gas permeation in graphene oxide-polymer self-assembled multilayers. *ACS Appl*. *Mater*. *Interfaces* **10**, 11242-11250, (2018).
